# Spatial transcriptomic profiling of isolated microregions in tissue sections utilizing laser-induced forward transfer

**DOI:** 10.1371/journal.pone.0305977

**Published:** 2024-07-25

**Authors:** Kaiqiang Ye, Wanqing Chang, Jitao Xu, Yunxia Guo, Qingyang Qin, Kaitong Dang, Xiaofeng Han, Xiaolei Zhu, Qinyu Ge, Qiannan Cui, Yun Xu, Xiangwei Zhao

**Affiliations:** 1 State Key Laboratory of Digital Medical Engineering, School of Biological Science & Medical Engineering, Southeast University, Nanjing, Jiangsu, China; 2 Department of Neurology, Nanjing Drum Tower Hospital, Affiliated Hospital of Medical School, Nanjing University, Nanjing, Jiangsu, China; Nathan S Kline Institute, UNITED STATES

## Abstract

Profiling gene expression while preserving cell locations aids in the comprehensive understanding of cell fates in multicellular organisms. However, simple and flexible isolation of microregions of interest (mROIs) for spatial transcriptomics is still challenging. We present a laser-induced forward transfer (LIFT)-based method combined with a full-length mRNA-sequencing protocol (LIFT-seq) for profiling region-specific tissues. LIFT-seq demonstrated that mROIs from two adjacent sections could reliably and sensitively detect and display gene expression. In addition, LIFT-seq can identify region-specific mROIs in the mouse cortex and hippocampus. Finally, LIFT-seq identified marker genes in different layers of the cortex with very similar expression patterns. These genes were then validated using in situ hybridization (ISH) results. Therefore, LIFT-seq will be a valuable and efficient technique for profiling the spatial transcriptome in various tissues.

## Introduction

Single-cell mRNA profiling can provide detailed transcriptomic data about organs and the main factors determining cell states [1]. However, in multicellular organisms, cell fates are strongly influenced by their local microenvironments [2]. Despite advances in massively parallel single-cell RNA sequencing (scRNA-seq), spatial information remains lost as cells dissociate. Spatial transcriptomics can provide novel insights into embryonic development [3], histopathology [4], neuronal study [5], brain disease [6], and tumor microenvironment [7]. Therefore, spatially resolved transcriptomics is critical for determining the molecular architecture.

Several methods have been developed for extracting spatial transcriptomes from cells. Early methods based on fluorescent in situ hybridization (FISH) could only find genes in specific subcellular regions [8, 9], but they were difficult to understand and time-consuming to perform. New techniques using barcoded solid-phase RNA capture, such as spatial transcriptome (ST) [10], slide-seq [11], and high-definition spatial transcriptome (HDST) [12], have enabled gene expression mapping on tissue sections. The expression profile of each pixel in the deterministic barcoding in tissue (DBiT) method [13] may only represent the average information of nearby cells because the pixels are randomly distributed throughout the tissue in a matrix. Even though these methods allow for whole-sample expression mapping and high-throughput spatial transcriptomic analysis, cells between capture units were missed.

By simply selecting microregions of interest (mROI), laser capture microscopy (LCM) is a commonly used technique for accurately targeting and isolating cells or tissue regions of interest using ultraviolet (UV) or infrared (IR) beams in tissue sections for scRNA-seq [14, 15]. However, the UV beam in LCM can cause tissue burning and damage to nucleic acid molecules [15]. In addition, using IR-based LCM results in cross-contamination of targeted and nontargeted samples [16].

Therefore, the prerequisite for spatial transcriptomics, particularly for rare tissue or cells, is the precise isolation of cells while minimizing the damage to nucleic acid molecules during operation. Laser-induced forward transfer (LIFT) is a direct printing technique that uses a pulsed laser beam to propel material from a donor thin layer to the receiving devices [17]. LIFT is a nozzle-free and noncontact technique with few limitations on the materials that can be printed; thus, LIFT-based methods have been successfully applied to various bioprinting processes (such as DNA, peptides, and proteins), with LIFT demonstrating excellent protection of the transferred biomolecules [18–21]. Many studies have shown that LIFT is viable for cell printing in cell biology and tissue engineering without affecting cell behavior or intracellular functions [22–25]. Specifically, unlike LCM’s UV beam, the near-infrared laser beam used in LIFT caused no damage to nucleic acids [15]. However, it did not affect the integrity and capability of selectively hybridizing transferred DNA [18, 26]. Thus, due to its high resolution and protection in bioprinting applications, the LIFT-based method is an effective technique for transferring a microregion of tissues for transcriptome analysis.

We present a LIFT-based isolation method combined with full-length mRNA sequencing (LIFT-seq) to detect gene expressions in mROIs (1–5 cells) isolated from tissue sections. To prepare the sequencing library, a picosecond pulsed near-infrared (NIR) laser (1064 nm) was focused on a glass slide containing an indium tin oxide (ITO) layer. The tissue samples were separated by vaporizing the induced layer. LIFT-seq was tested for sensitivity and reproducibility in mouse brain sections, and region-specific genes were identified in the cortex and hippocampus. Finally, layer-specific genes were identified in cortex subregions and validated in the Allen brain atlas, indicating cell heterogeneity between adjacent regions despite highly similar gene expression patterns.

## Materials and methods

### Assembly and operation of LIFT device

**Fig 1:**
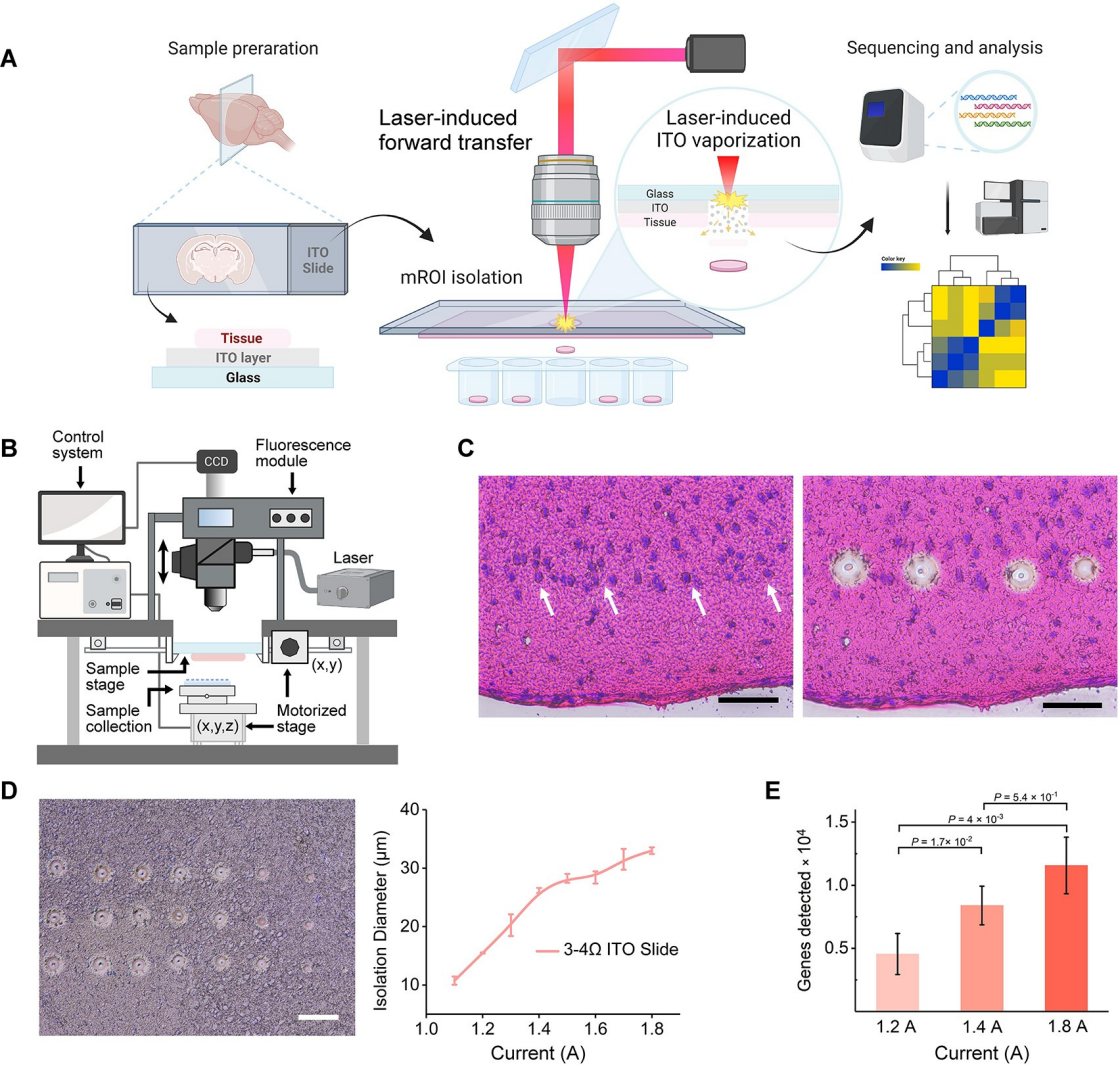
LIFT-based isolation coupled with a full-length mRNA-sequencing protocol (LIFT-seq) for profiling mROI gene expressions from tissue sections. (A) Workflow of LIFT-seq and a schematic of LIFT-based isolation: an IR laser vaporized a solid ITO thin layer, which served as a pushing force to separate microregion tissues from the section. (B) The key components of the LIFT-based isolation system. (C) Microscopic observations of H&E-stained mouse brain sections prior to and following LIFT isolation. Scale bar: 50 μm. (D) Size-controlling analysis of LIFT isolation on ITO-coated slides with a surface resistivity of 3–4 Ω per square (n = 3). (E) The average number of genes detected in mouse brain sections at different laser powers. The threshold for gene detection was set to ≥ 1 (n = 4).

The framework (Fig 1B) shows that the LIFT instrument was built on a custom-designed optical microscope platform that included a fluorescence module, focus control unit, sample placement, and collection stages. A picosecond pulsed laser with a wavelength of 1064 nm was added to the optical path and controlled by the shutter. The instrument’s modules were tailored to the system, and the control system was implemented using in-house software. When using LIFT, a tissue section attached to an ITO-coated slide was placed on the sample stage, and the focus control was adjusted to obtain a clear image for targeting mROI. An objective lens scanned the entire section to create a navigation image. The targeted mROI was moved to the laser spot location using the scanned image as a guide, and the collection tube was precisely placed beneath it. Then, the shutter was closed and the laser device’s current intensity was adjusted before reopening it to isolate the mROI. The size of the isolated tissues could be controlled by modulating the intensity of the current and the objective lenses at different magnifications. The isolated mROI was collected in a cap containing lysis buffer and immediately centrifuged at the bottom of a collection tube. This tube could be stored at -80°C until the sequencing library was prepared.

### Reagents and materials

All the reagents used in the library preparation experiment were molecular biology grade and certified as nuclease-free as possible. They were prepared with nuclease-free water. The materials were either certified nuclease-free or treated in-house with Surface RNase Erasol. The instruments and work surfaces were cleaned with 75% ethanol and treated with RNaseZAP while the environment was sterilized with UV light.

### Preparation of animals and tissue sections

The Ethics Committee of Zhongda Hospital Southeast University approved the animals’ care and use in this study. Eight-week-old male C57Bl/6J mice were acquired from Shanghai Southern Model Biotechnology Co., Ltd. They were anesthetized with tribromoethanol (500 mg/kg) (Sigma, Saint Louis, USA) and then killed via cervical dislocation. The brain was quickly dissected from a sacrificed mouse, lightly washed with PBS, and embedded in a pre-cooled optimal cutting temperature (OCT) embedding medium (SAKURA) before being flash-frozen in liquid nitrogen to preserve its morphology and mRNA quality. A rabbit’s small vessel and a rat’s peripheral nerve were dissected from sacrificed animals, fixed in formalin overnight, and embedded in OCT before being frozen. For the library preparation experiment, ITO slides were washed three times with pure ethanol, cleaned with RNaseZAP (Invitrogen) for 10 min, and thoroughly rinsed with deionized water before being stored in tissue section slide boxes. After achieving temperature equilibrium, a 10 μm section was prepared onto an ITO slide and stored at -80°C for further processing. Fresh frozen sections were fixed in a 4% (w/w) formaldehyde solution (diluted in PBS) for 10 min before being washed with 1xPBS and dehydrated with isopropanol. Fixed sections were stained with hematoxylin, bluing buffer, and eosin, as directed by the manufacturer (Solarbio). Finally, the sections were allowed to air-dry at room temperature. Fresh frozen sections were dehydrated with a series of ethanol solutions (100%, 95%, and 70%, v/v) for 30 s, then stained with 0.5% (w/v) cresyl violet solution (diluted in 70% ethanol) for 1 min, dehydrated with pure ethanol, and stored at -80°C until LIFT isolation.

### LIFT isolation from different kinds of samples

A hot 0.5% (w/v) agarose solution diluted in water was pipetted onto an ITO slide to ensure that LIFT-based isolation was effective. After cooling at room temperature, the slide formed a film of approximately 50 μm thickness. The agarose-coated ITO slide was placed on the sample stage and subjected to LIFT isolation with current intensities of 1.4 A and 1.8 A using the laser device. Following isolation, the agarose film and ITO layer states were examined using SEM. For bright-field isolation, samples from various tissues were H&E stained and LIFT isolated. Images were taken before and after isolation using a CCD camera or SEM to investigate the results. A DAPI-stained mouse brain section was used for fluorescent isolation, and the fluorescence module’s channel was modified to provide excitation light for exciting the fluorescent dye while filtering the emission light for imaging (Fig 1B).

### Size control of LIFT isolation

ITO slides with different surface resistivities were prepared to investigate the control of isolation size, and mouse brain tissue was sectioned onto them. LIFT isolation was achieved using a 10x objective lens (OLYMPUS NA: 0.30). All sections were stained with cresyl violet as described above. Different current intensities of the laser device were adjusted to control the size of LIFT isolation: from 1.1 A to 1.8 A (n = 3) for 3–4 Ω ITO slides, and from 1.4 A to 2.0 A (n = 3) for 7–10 Ω and 10–15 Ω ITO slides. The diameters of these isolation defects were measured and calculated following isolation.

### LIFT isolation and collection for sequencing

When the LIFT instrument’s entire system was successfully tested, a mouse brain section was placed in the sample stage holder. Sections were stained with cresyl violet to identify anatomical structures and isolate mROIs from the hippocampus and cortex regions. mROIs were isolated from three layers of the cortex, evenly distributed from outer to interior. A row of 200 μl PCR tubes was placed in the collection stage, with the inner caps facing upward. The procedure for isolating mROIs from tissue sections into a PCR tube took 1–3 min per mROI. To extract mRNA with high sensitivity, we decreased the volume of the lysis solution. Each cap was filled with 2.75 μl lysis solution (Single Cell Lysis kit, Life Technologies). To collect samples, 25-μm-diameter mROIs were placed in caps and centrifuged for 10 s. Before collection, all samples were placed in an ice box to avoid mRNA degradation. After LIFT isolation, samples were stored at -80°C while reagents were prepared for the following steps.

### cDNA and sequencing library preparation

The samples were subjected to direct lysis, and the library was prepared for sequencing using the improved ultralow RNA-seq library preparation protocol based on the previous study’s SS2 protocol [27, 28].

If ice was available, each of the following procedures was performed. The samples were spun down again and placed in a lysis solution at 25°C for 10 min to extract the mRNA. Following that, 0.25 μl of stop solution (Single Cell Lysis Kit, Life Technologies) was added to each sample and left to sit at 25°C for another 2 min. Before starting the reverse transcription (RT) reaction, 1 μl 10mμ dNTP mix, 1 μl 10 uμ oligo-dT primer (Sangon, 5′-AAGCAGTGGTATCAACGCAGAGTACT30VN-3′), and 0.25 μl RNAse inhibitor (40 U μl^− 1^, Takara) were added into the sample. The sample was incubated for 3 min at 72°C to open the RNA’s secondary structure. The samples were placed on ice immediately afterward. The following mixture was added to the sample: 2.5 μl 5× first-strand buffer, 0.1 μl 100 μμ LNA-TSO primer (Sangon, 5′-AAGCAGTGGTATC AACGCAGAGTACATrGrG+G-3′), 1 μl 25 mμ MgCl2 (Sigma-Aldrich), 0.5 μl 100 mμ DTT, 2 μl 5 μ betaine (Sigma-Aldrich), 0.25 μl 40 U μl^− 1^ RNAse inhibitor, and 1μl 200 U μl^− 1^ Maxima H minus reverse transcriptase (Thermo Fisher). The samples were subjected to the following RT reactions in order: 90 min at 42°C, 10 cycles of (50°C for 2 min and 42°C for 2 min), and 5 min at 85°C. For pre-amplification, 12.5 μl 2 × KAPA HiFi HotStart Ready mix and 0.5 μl 10 μμ ISPCR primers (Sangon, [AmC6] 5′-AAGCAGTGGTATCAACGCAGAGT-3′) were added into the first strand cDNA. The PCR cycle was then completed in the following manner: 3 min at 98°C, then 25 cycles of (20 s at 98°C, 15 s at 67°C, and 6 min at 72°C). Following the manufacturer’s protocols, the samples were purified with Ampure XP beads and dissolved in 20 μl of nuclease-free water. The quantity was calculated using Qubit 4.0 with dsDNA HS Assay Kit (Life Technologies), and the fragment distribution was detected with an Agilent 4150 TapeStation.

For sequencing library generation, 5 ng of cDNA was pipetted into a new tube for tagmentation reaction with the One-Step DNA Lib Prep Kit for Illumina (ABclonal). The cDNA was fragmented during the transposase-based tagmentation reaction, which involved ligating the sequencing adaptors to both ends of the cDNA fragments. After tagmentation, the PCR reaction for index ligation and fragment enrichment was carried out using the following program: 3 min at 72°C, 45 s at 98°C, 13 cycles of (15 s at 98°C, 30 s at 60°C, and 3 min at 72°C), and 5 min at 72°C. Following bead purification, the amount and distribution of cDNA fragments were determined. The cDNA library was sequenced using the HiSeq X10 PE150 sequencer (Illumina, USA).

### Read mapping and data analysis

RNA-seq raw data were filtered to remove adapters, and poly-clean data were processed by FastQC software (v0.11.4) for quality control (QC) before being mapped to the mouse reference genome mm10 using Hisat2 software (version 2.2.1) with default parameters. HTSeq (version 1.99.2) was used to map reads to references, and the number of genes detected was calculated using counts ˃ 0. The read counts were converted to counts per million (CPM) and then normalized using edgeR’s CPM function with default parameters for further analysis [29, 30]. The top 1000 variable genes in expression were computed to compare the correlation of individual mROIs in iDEP (version 0.96) and perform heatmap/PCA/tSNE clustering [31]. Differential expression analysis (DESeq2) was performed using a 0.1 FDR cutoff [29]. The log_2_ normalized gene expression level was used to compare region- and layer-specific genes. We performed a gene ontology (GO) enrichment analysis on enriched gene sets among the 2000 most variable genes in iDEP. We presented the top five enriched terms with adjusted *P* values < 0.01 in BP, MF, and CC. We also used scMCA to investigate the cell-type enrichment of these differentially expressed genes [32–34]. ISH images of the identified marker genes were searched for and downloaded from the Allen brain atlas.

## Results

### Performance of LIFT-based isolation

Fig 1A depicts the LIFT-based isolation principle as well as the LIFT-seq workflow. A laser pulse vaporized an ITO-thin layer, which then served as a pushing force to separate micro-region tissues from the section. The sequenced reads were processed and analyzed after the isolated samples’ direct lysis and library preparation. The ability to move solid-phase material from the solid donor layer was initially tested on a 50-μm-thick agarose gel membrane coated on an ITO slide. An IR laser pulse caused an evident defect in SEM images (S1B Fig), indicating that the gel separated as expected. An H&E-stained mouse brain tissue section (Fig 1C) was prepared on ITO-coated slides to test LIFT performance in tissue sections. Following LIFT operation, four mROIs (2–3 cells) were isolated from the section observed under an optical microscope (Fig 1C). In addition, mROIs were successfully separated from H&E-stained sections of rat peripheral nerve (S1C Fig), rabbit small vessel (S1D Fig), and formalin-fixed paraffin-embedded (FFPE)- and 4’,6-diamidino-2-phenylindole (DAPI)-stained mouse brain sections (S1E and S1F Fig). To isolate specific-size tissues with precision and consistency, ITO slides with varying surface resistivities (Fig 1D, S2A and S2B Fig) were used to investigate the relationship between laser device current intensity and isolation diameters. Following LIFT isolation with various ITO slides and laser intensities, micrographs revealed defects with clear-sized profiles on the sections. Isolation diameters ranged from 5 μm to 35 μm, depending on the laser device’s current intensity. For larger-size isolation, an ITO slide with reduced surface resistivity is recommended. These findings show that LIFT is a viable method for isolating mROIs (down to the single cell level) from a wide range of tissue sections, with high stability and reproducibility.

### Validation of LIFT-seq in mouse brain tissues

The ability of LIFT-seq to recover mRNA from isolated tissues was tested on fresh frozen mouse brain sections using full-length mRNA-seq [27]. Different laser powers were used to isolate micro-region tissues from mouse brain sections randomly and test the feasibility of cDNA recovery and gene detection from such small amounts of tissue (down to the single-cell level). The isolated tissue was placed in a 200-μl PCR tube cap for direct lysis and library preparation. The yield and quality of cDNA products were assessed following reverse transcription and a 25-cycle amplification. As expected, the total cDNA yield increased when larger tissues were collected by increasing the power of the laser device (S3D Fig), and the high quality of cDNA profiles was comparable to each other (S3A–S3C Fig), indicating that nucleic acid integrity was not compromised during LIFT operation, which is critical for full-length sequencing library preparation and detailed analysis. On average, over 4,000 genes (n = 4) were detected from ~15 μm-size mROI (Fig 1E, 1.4 A current intensity) and over 10,000 genes (n = 5) from ~30 μm-size mROI (Fig 1E and 1.8 A current intensity). The consistent results demonstrated that LIFT-seq can efficiently identify genes in mROIs containing multiple or even a single cell and recover high-quality cDNA.

**Fig 2:**
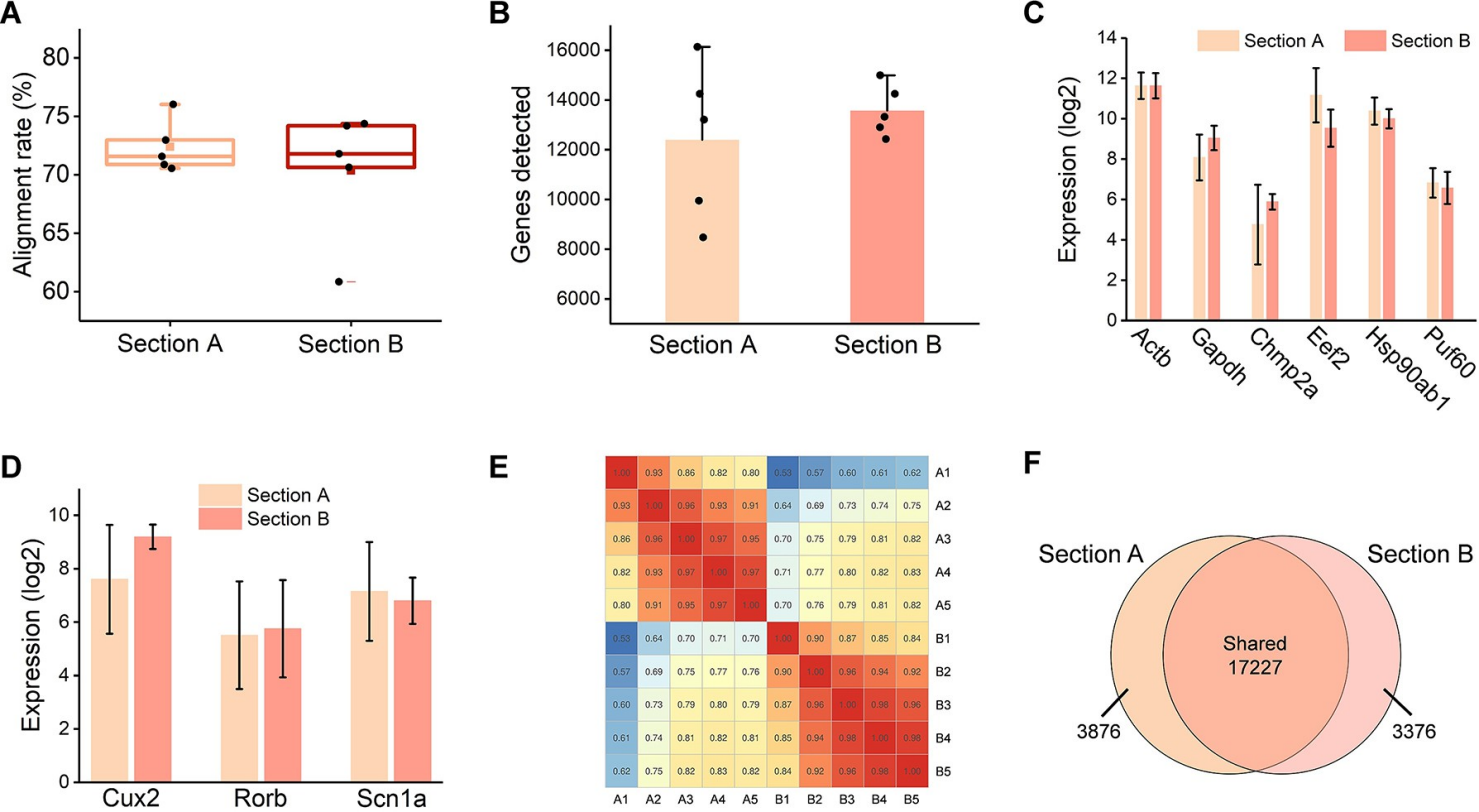
LIFT-seq revealed similar gene expression profiles in the same positions in two adjacent sections. (A) Alignment rate of mROIs isolated from the two sections. (B) The average number of genes found in mROIs isolated from both sections. (C) The expression of housekeeping genes in isolated mROIs (n = 5). (D) Region-specific genes were found in both sections at comparable expression levels (n = 5). (E) As the mROIs between the two sections increased, so did the gene expression correlation. (F) The Venn diagram shows that sections A and B shared 17,227 genes (approximately 83%).

To ensure that LIFT-seq is a reliable tool for resolving transcriptomes from specific micro-region samples, ten (five per section) mROIs were collected from the same position in the cortex region of two adjacent sections, and sequencing libraries were prepared afterward. Following data analysis, an average of 8,989 genes were identified at a depth of 5 million reads per sample, and the alignment rate and gene detection plots for two sections were shown (Fig 2A and 2B). Furthermore, the expression levels of some housekeeping genes in the mROIs from both sections were comparable. Genes such as *Scn1a*, *Rorb*, and *Cux2* were found in both groups and expressed similarly (Fig 2D) [35–38]. Thus, we assumed that samples from the same position in two adjacent sections would have similar expression profiles. The RNA-seq data for each mROI in sections A and B were sorted and analyzed. We discovered that gene expression correlation increased with increasing mROIs, indicating a similar gene expression pattern between the two groups (Fig 2E). The Venn diagram showed that 17,227 genes were present in both sections A and B, accounting for approximately 83% (Fig 2F).

### LIFT-seq profiling of gene expressions in the cortex and hippocampus regions

**Fig 3:**
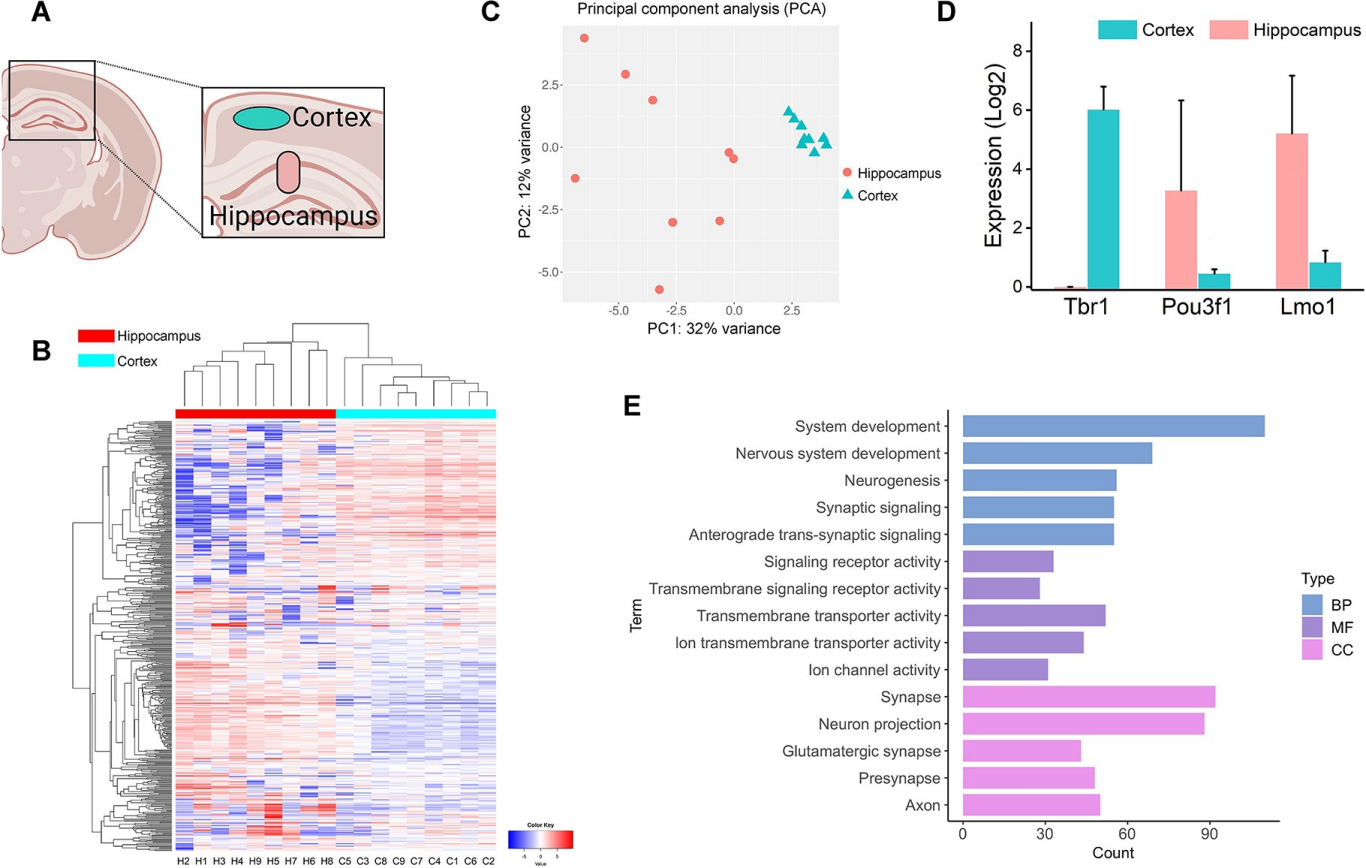
LIFT-seq can profile gene expressions in region-specific mROIs derived from the cortex and hippocampus regions. (A) LIFT-seq-isolated mROIs from the mouse brain cortex and hippocampus. (B) Clustering these mROIs from various regions using a heatmap analysis. (C) PCA clustering of these mROIs. (D) Expression of marker genes in cortex and hippocampus. (E) The top five terms enriched in BP, MF, and CC based on GO analysis. System development, Nervous system development, Neurogenesis, Signaling receptor activity, and Transmembrane signaling receptor activity terms were enriched from genes upregulated in the cortex. Anterograde trans-synaptic signaling, Synaptic signaling, Transmembrane transporter activity, Ion transmembrane transporter activity, Ion channel activity, Synapse, Neuron projection, Presynapse, Glutamatergic synapse, and Axon terms were enriched from genes upregulated in the hippocampus.

We tested LIFT-seq on the mouse brain’s cortex and hippocampus to see if it could profile gene expression of region-specific mROIs (Fig 3A). We identified the two areas by looking at the stained anatomical structures in the tissue section. Then, nine mROIs from each area—18 in total—were extracted from the cortex and hippocampus and stored in a library. We used ng and heatmap/PCA clustering based on variable genes (Fig 3B and 3C). We found a clear separation between these mROIs from different regions, indicating that mROIs in the same brain region will have similar gene expression patterns. Thanks to cell typing studies in the mouse cortex and hippocampus [36], we could count the marker genes expressed in these mROIs. *Tbr1*, a marker gene for the cortex, was only detected in mROIs from the cortex region. Meanwhile, the hippocampal marker genes (*Pou3f1* and *Lmo1*) were expressed at significantly high levels in hippocampal mROIs (Fig 3D). GO enrichment was performed based on differential genes among these mROIs. The five most used terms in biological process (BP), molecular function (MF), and cellular component (CC) were found to be strongly related to nervous system activities, processes, and functions (Fig 3E). When expression correlations between individual mROIs were compared, those from the cortex region were more similar than those from the hippocampus region (S4C Fig), indicating that these mROIs are spatially heterogeneously distributed. LIFT-seq’s ability to characterize gene expressions in regional mROIs was validated.

### LIFT-seq enables the profiling of cortex sub-regions

**Fig 4:**
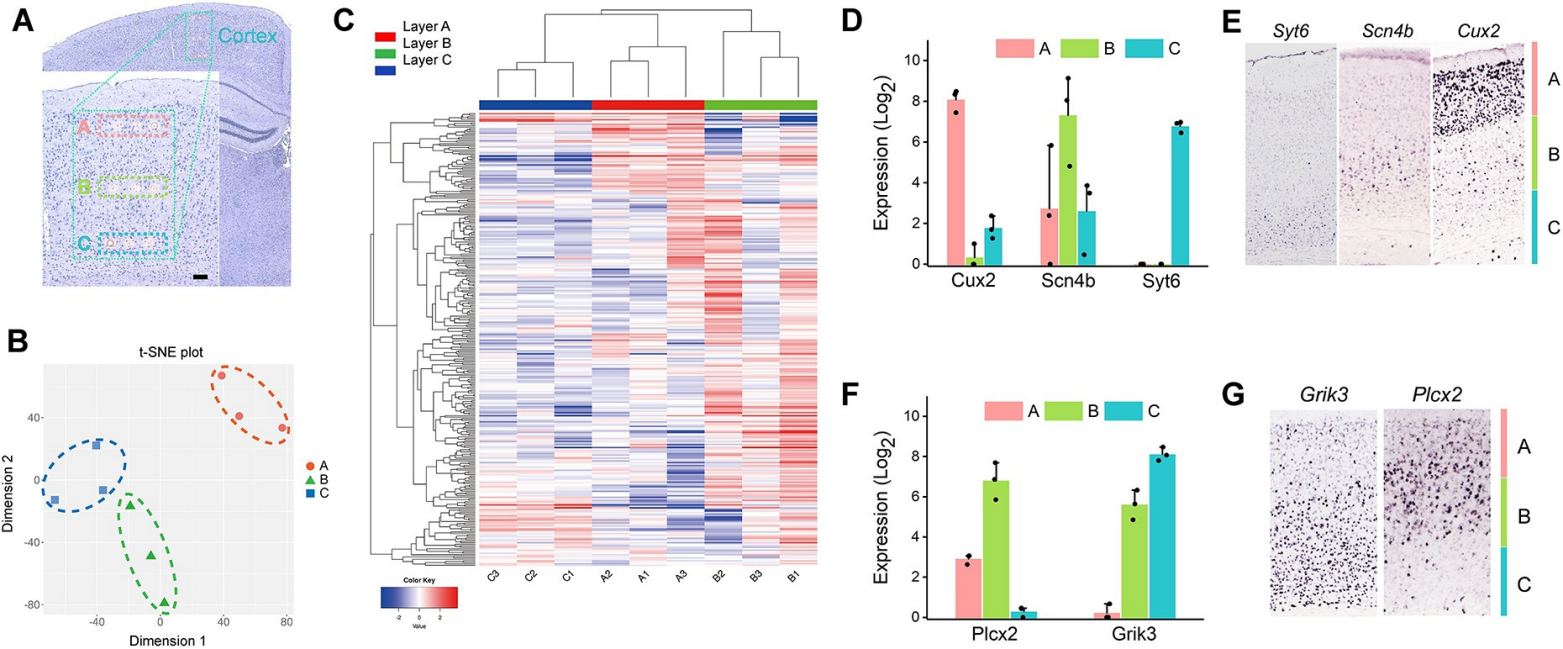
LIFT-seq uncovered layer-specific genes in the mouse brain’s cortex region. (A) Magnified image of mROIs isolated in layers A, B, and C of the cortex. (B) tSNE shows clear distinctions between clusters of layer-specific mROIs. (C) Heatmap clustering shows that mROIs isolated from the same layers have similar gene expression profiles. (D) The expression of layer-specific genes in each layer. (E) The Allen Brain Atlas’ ISH results were used to identify layer-specific genes. (F) Expression levels of genes identified in adjacent layers. (G) ISH images show layer-specific expressions in layers AB and BC.

We extracted layer-specific mROIs from three layers (A, B, and C) of the cortex region (Fig 4A) to demonstrate how LIFT-seq can characterize gene expression in sub-regions. Layer-specific mROIs were classified into three groups using tSNE and heatmap analysis based on transcriptome profiles (Fig 4B and 4C). These findings suggested that the cortex could be divided into outer-to-inner layers. To back this up, we compared the differential genes layer by layer to identify potential marker genes for each layer (S5B Fig). *Cux2*, *Scn4b*, and *Syt6* were designated layer-specific genes, marking layers A, B, and C respectively. Their expression levels varied across different areas (Fig 4D). The Allen Brain Atlas ISH results (Fig 4E) revealed the layer-specific mappings of these marker genes, which were well-defined and closely related to their expression level. The genes that marked adjacent layers (*Plcx2* marked layers A and B, *Grik3* marked layers B and C) were also identified and visualized in ISH images (Fig 4F). Based on our observations of layer-specific subdivisions in the cortex, we confirmed that LIFT-seq can be used to identify microstructures with region-specific gene expression patterns.

## Discussion

Spatial transcriptomics has been shown to provide an overview of gene expression while also mapping the molecular topography across tissues [39–41]. To profile site-specific samples with full-length mRNA recovery, we need region-isolation methods and Smart protocols that switch mechanisms at the 5′ end of the RNA template [42]. For example, Smart-Seq2 (SS2), the gold standard in recent years, enables a much more in-depth transcriptome analysis with high sensitivity and single-cell resolution [43].

This study describes LIFT-seq, a full-length mRNA-seq method capable of spatially characterizing gene expression from mROIs with high sensitivity and single-cell resolution. Unlike microneedle-based methods, which make it difficult to create stable tips for isolating samples at the single-cell level [44–46], laser-based methods can shape the laser beam within a few microns for high-resolution capture. In LIFT-seq, an infrared (1064 nm) laser was used to protect mRNA molecules from UV damage in regular LCM [15]. Moreover, the laser energy used to protect the mRNA was low because the ITO layer instantly evaporates when exposed to the laser (as confirmed by a 1/50-second laser shutter setting). Furthermore, LIFT-seq demonstrated robustness and flexibility in isolating different types of samples without changing the parameters, and the controllable size of the isolated samples is determined by the current intensity or objective lens. Notably, LIFT-seq demonstrated high collecting rate efficiency without necessitating complex tuning before and during isolation. It is helpful for quickly collecting mRNA and preventing it from degrading. Consequently, LIFT-seq can extract high-quality cDNA from separated samples (S3A–S3D Fig), allowing researchers to create libraries ready for sequencing and gene discovery.

The mouse brain is made up of many different types of specialized cells that are spatially organized into anatomical structures [47]. scRNA-seq has been used on type cells in various parts of the mouse brain, but information about each cell’s location was lost when the tissue was broken down to make cell suspension [35, 36, 38]. Only with anatomical resolution can cell types and marker genes be identified [36]. Using LIFT-seq on mouse brain sections, mROIs from different brain regions were divided into region- and subregion-defined clusters using heatmaps and tSNE/PCA analyses. Moreover, *Tbr1* was identified as a marker gene for all pyramidal cells in the mouse somatosensory cortex (S1) by comparing its gene expression to that of cells in the hippocampus region [36]. Based on this, we tested whether LIFT-seq could identify *Tbr1* in mROIs isolated from the cortex region. Remarkably, *Tbr1* was uniquely expressed in all mROIs in the cortex at high levels (Fig 3D). *Pou3f1* and *Lmo1* also confirmed the consistent findings. Genes found in the hippocampus showed significantly higher expression levels in mROIs (Fig 3D) (P < 0.05). Based on these findings, LIFT-seq can profile the expression of region-specific genes at a single-cell level.

We discovered that the correlation of gene expression in cortical mROIs is similar, but hippocampus mROIs differ significantly (S4C Fig). We used LIFT-seq to identify layer-specific mROIs in three mouse cortex layers (A, B, and C) (Fig 4A) to demonstrate its effectiveness and determine whether it could distinguish between mROIs with similar expression patterns. Heatmap/tSNE’s hierarchical cluster results show these mROIs on a larger scale, indicating that the cortex region is divided into layers, with the outer layers being denser than the inner layers (Fig 4B and 4C). When looking at the layer-determined clusters of isolated mROIs, we considered the possibility of identifying layer-specific genes. As expected, differentially expressed genes found primarily in one layer (Fig 4D) were used to identify genes marked by each layer. The housekeeping genes expressed in each layer were also identified (S5A Fig). Additionally, we examined the layer-specific genes using ISH results, which revealed excellent layer-specific topographies corresponding to gene expression patterns (Fig 4E). LIFT-seq is generally sensitive enough to determine gene expression in highly similar or rare samples.

## Conclusions

In summary, LIFT-seq was presented, a full-length mRNA-seq method capable of spatially profiling gene expression with high resolution and sensitivity. LIFT-seq’s flexibility made it a viable option for quickly and precisely capturing mROIs from rare samples for further analysis. Consequently, LIFT-seq will help characterize the spatial transcriptome and other spatial-based omics.

## Supporting information

S1 File (DOCX)

S1 Fig (TIF)

S2 Fig (TIF)

S3 Fig (TIF)

S4 Fig (TIF)

S5 Fig (TIF)
