# Supplementary material for: Spatial transcriptomic profiling of isolated microregions in tissue sections utilizing laser-induced forward transfer
Source: PLoS One. 2024 Jul 25;19(7):e0305977. doi: 10.1371/journal.pone.0305977 (PMC11271912; doi:10.1371/journal.pone.0305977)
Supplement: S1 File — (DOCX) [file pone.0305977.s001.docx]

Spatial transcriptomic profiling of isolated microregions in tissue section utilizing laser-induced forward transfer

Kaiqiang Ye^1^, Wanqing Chang^1^, Jitao Xu^1^, Yunxia Guo^1^, Qingyang Qin^1^, Kaitong Dang^1^, Xiaofeng Han^1^, Xiaolei Zhu^2^, Qinyu Ge^1^, Qiannan Cui^1^, Yun Xu^2^, Xiangwei Zhao^1^*

^1^ State Key Laboratory of Digital Medical Engineering, School of Biological Science & Medical Engineering, Southeast University, Nanjing, Jiangsu, China

^2^ Department of Neurology, Nanjing Drum Tower Hospital, Affiliated Hospital of Medical School, Nanjing University, Nanjing, Jiangsu, China

* Corresponding author

E-mail: [xwzhao@seu.edu.cn](mailto:xwzhao@seu.edu.cn) (XZ)

**
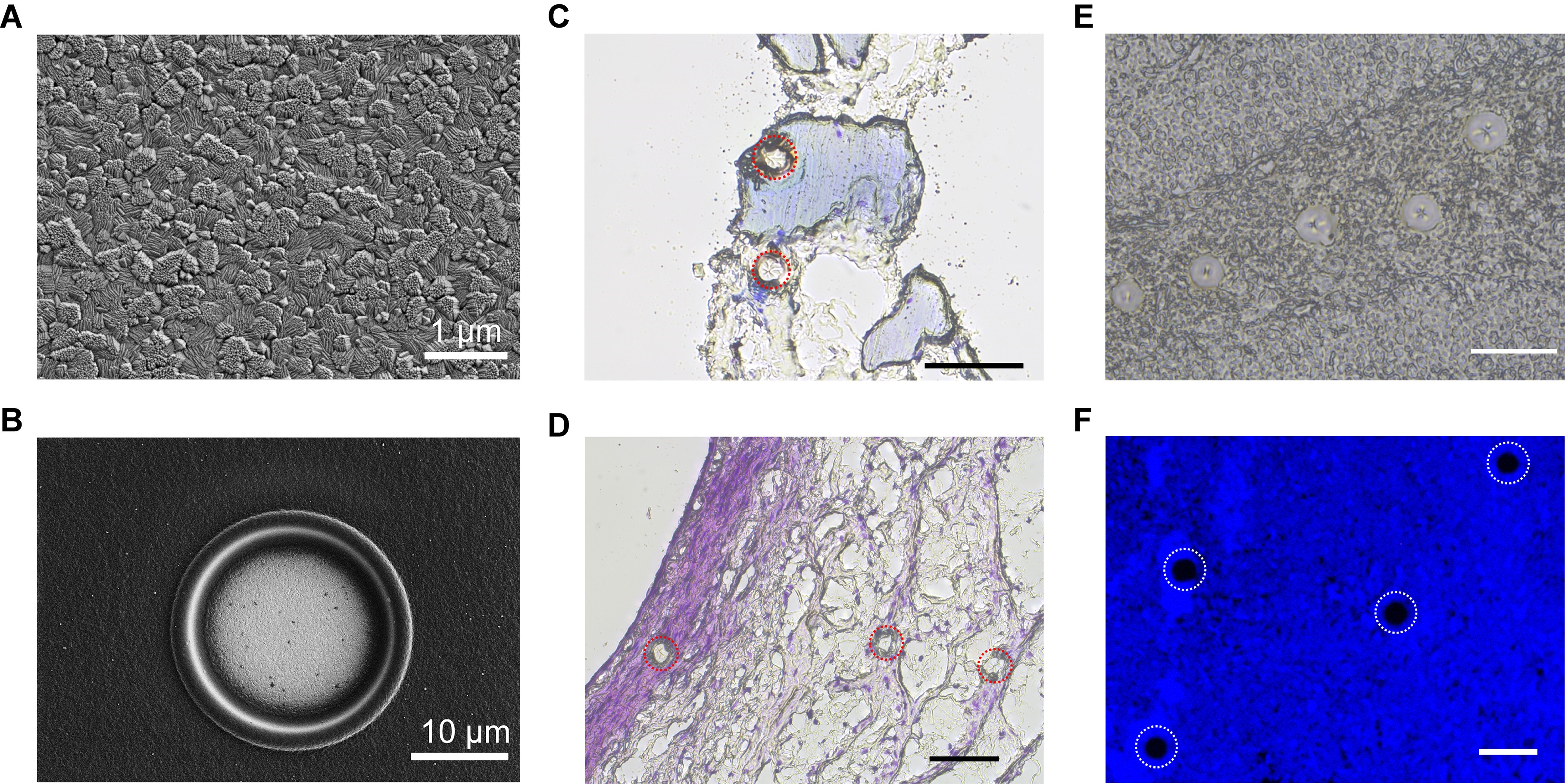
S1 Fig**. **Validation of LIFT-based isolation in different types of tissue sections.** (A) SEM observation on the surface of the ITO layer. (B) SEM observation after LIFT isolation in a 50-μm-thick agarose gel membrane. LIFT isolation on (C) rat peripheral nerve sections, (D) H&E stained rabbit small vessel section, (E) formalin-fixed paraffin-embedded mouse brain section, and (F) DAPI stained mouse brain section. Scale bar: 50 μm.


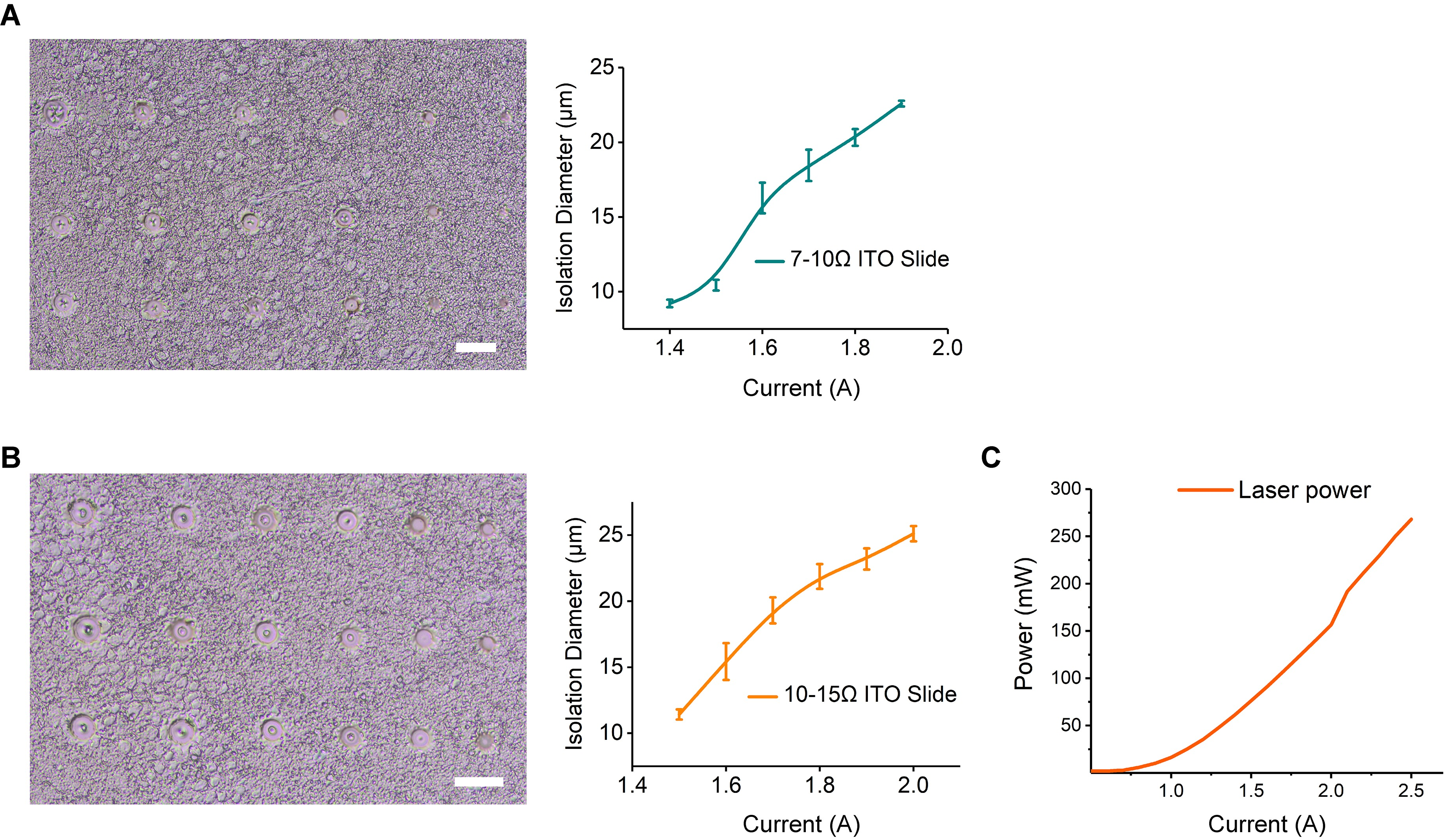


**S2 Fig. Size investigation of LIFT isolation within tissue sections.** Morphology observation and measured diameters after LIFT isolation on ITO coated slides with a surface resistivity of (A) 7-10Ω and (B) 10-15Ω per square (n = 3). (C) The laser output varied with the intensity of the current measured beneath a 10x objective lens (OLYMPUS, NA: 0.30).

**
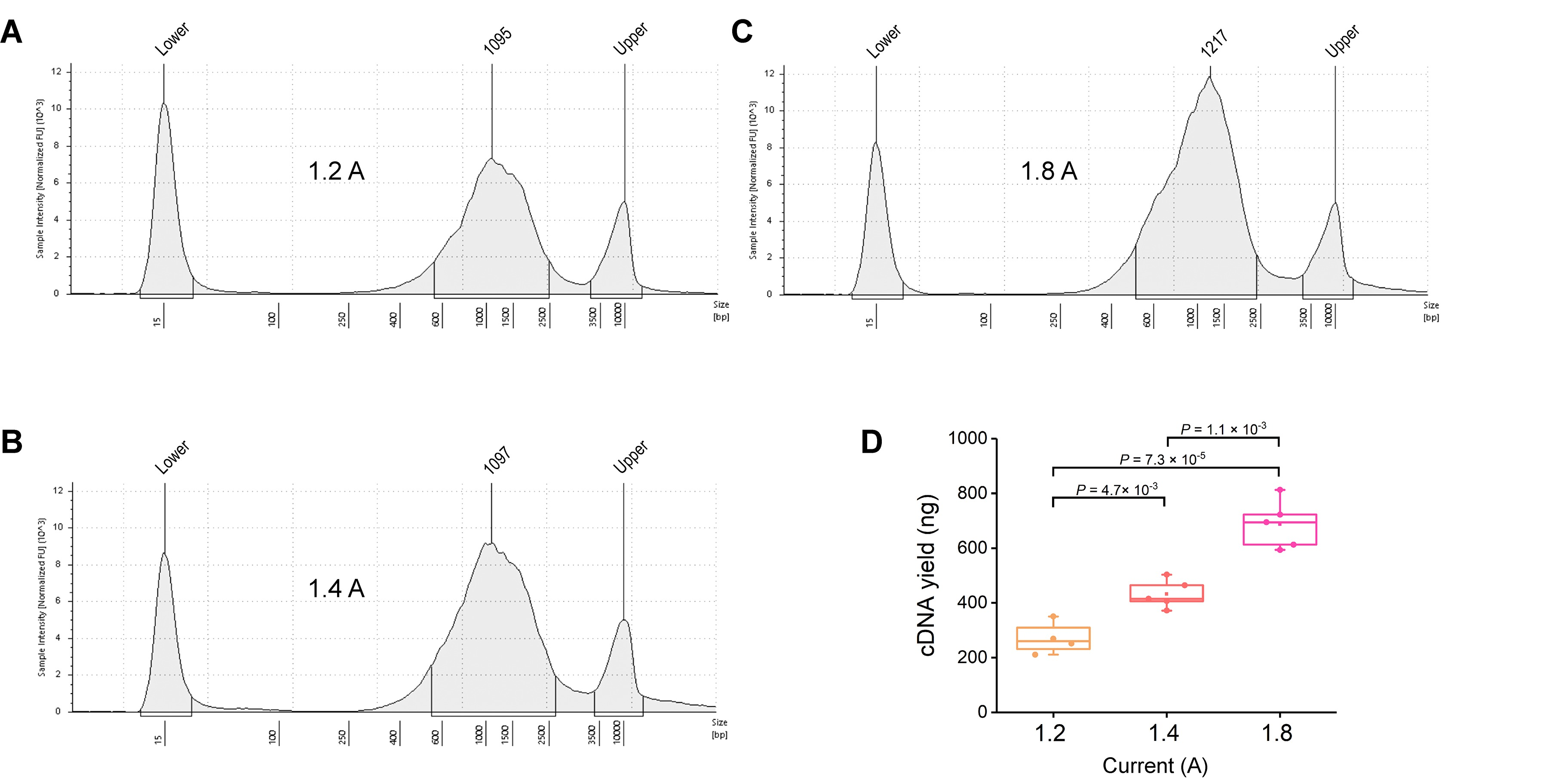
**


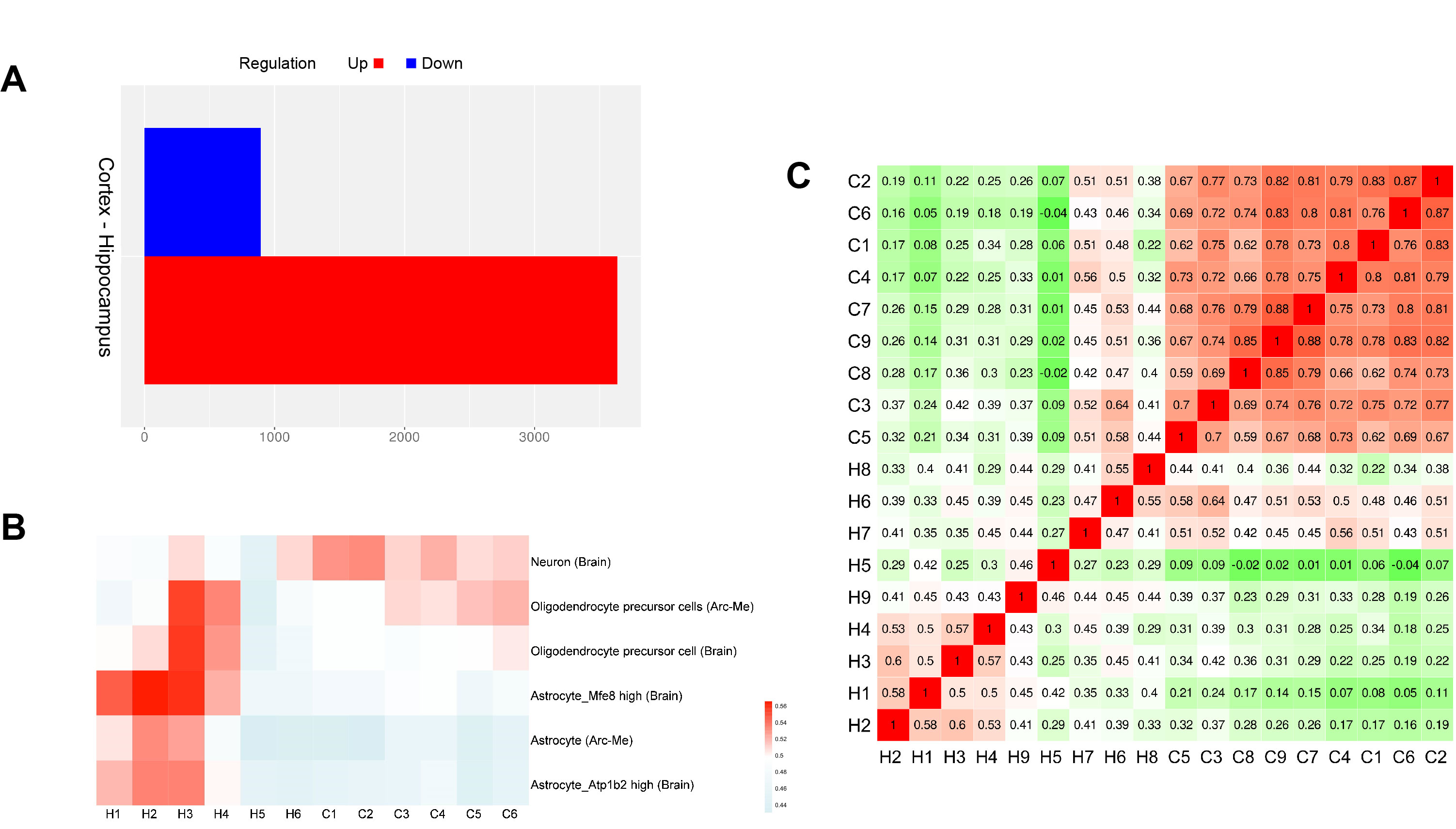
**S3 Fig.** **Quality of cDNA retrieved from isolated mROIs.** (A-C) The bioanalyzer profiles of cDNA libraries generated from three laser powers were of comparable quality. (D) cDNA yield varied with increasing laser power as a result of larger mROIs being isolated.

**S4 Fig. Comparison of gene expression in different regions and correlations between individual mROI from mouse brain section.** (A) The number of genes with differential expression generated by comparing mROIs from the cortex and hippocampus. (B) The cell-type enrichment of these differentially expressed genes using scMCA analysis. (C) Individual mROI correlations revealed more similar gene expression patterns in the cortex region than in the hippocampus region.


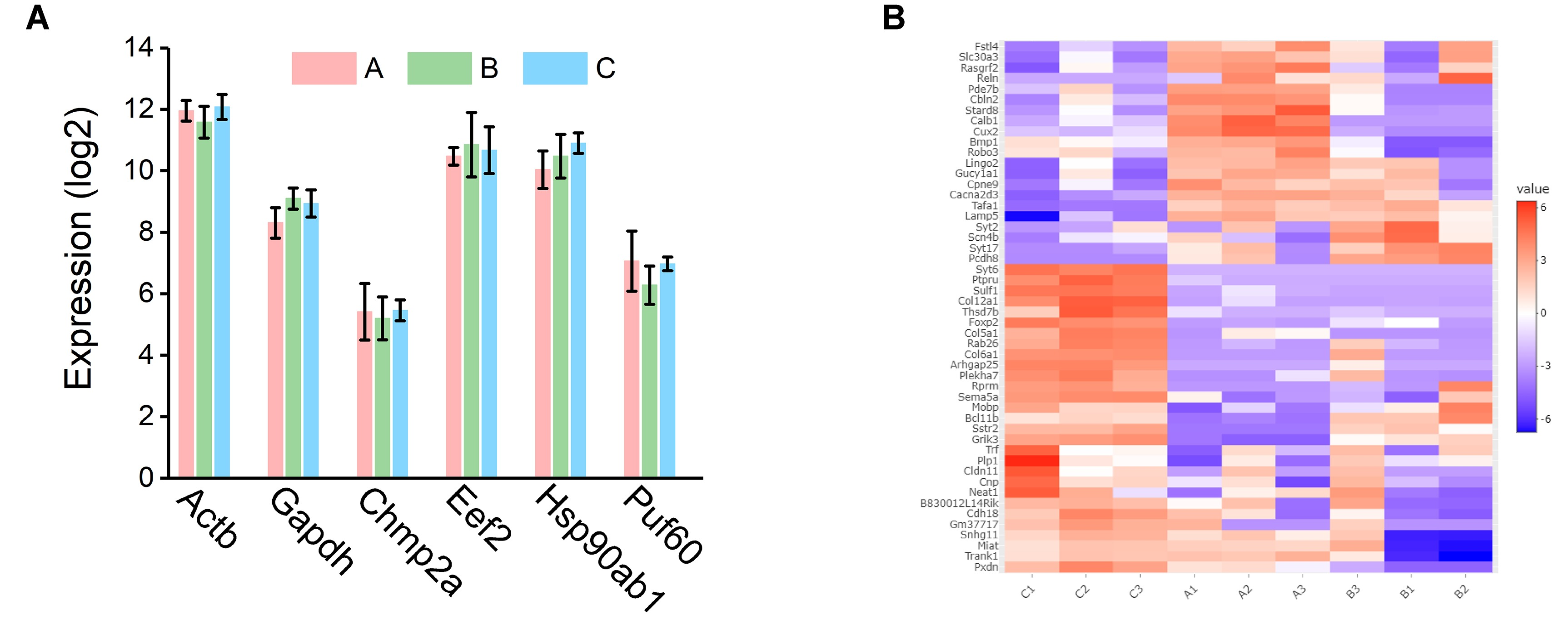


**S5 Fig.** **Expression levels and differential genes from three layers.** (A) Expression levels of housekeeping genes in isolated mROIs from three layers. (B) Top 50 differential genes by comparing mROIs from the three layers.

| Reagents | Source | Identifier | Size | Price（USD） | Cost per sample |
| --- | --- | --- | --- | --- | --- |
| Single cell lysisi kit | Invitrogen | 4458235 | 100 preps | 943.5 | 2.35 |
| dNTP mix | Vazyme | P031-01 | 1 ML | 17.1 | 0.017 |
| DTT | Sigma-Aldrich | 646563 | 10 x 0.5 ML | 119.73 | 0.0012 |
| RNAse inhibitor | Takara | 2313A | 5,000U | 37.3 | 0.15 |
| MgCl2 | Sigma-Aldrich | M1028 | 10 x 1 ML | 9.14 | 0.009 |
| Betaine | Sigma-Aldrich | B0300 | 1.5 ML | 35.68 | 0.0476 |
| Maxima H minus reverse transcriptase | Thermo Scientific | EP0752 | 10,000 | 174.87 | 3.5 |
| KAPA HiFi HotStart Ready mix | Roche | KK2602 | 6.25 ML | 238.9 | 0.478 |
| One-step DNA Lib Prep Kit for Illumina | ABclonal | RK20238 | 96 RXN | 2304 | 24 |
| Primer | Sangon | NA | NA | NA | 2.13 |
| Total | 32.683 | | | | |

**S1 Table. Key reagents list for library preparation.**
