# Supplementary figures and images for: Spatial transcriptomic profiling of isolated microregions in tissue sections utilizing laser-induced forward transfer
Source: PLoS One. 2024 Jul 25;19(7):e0305977. doi: 10.1371/journal.pone.0305977 (PMC11271912; doi:10.1371/journal.pone.0305977)

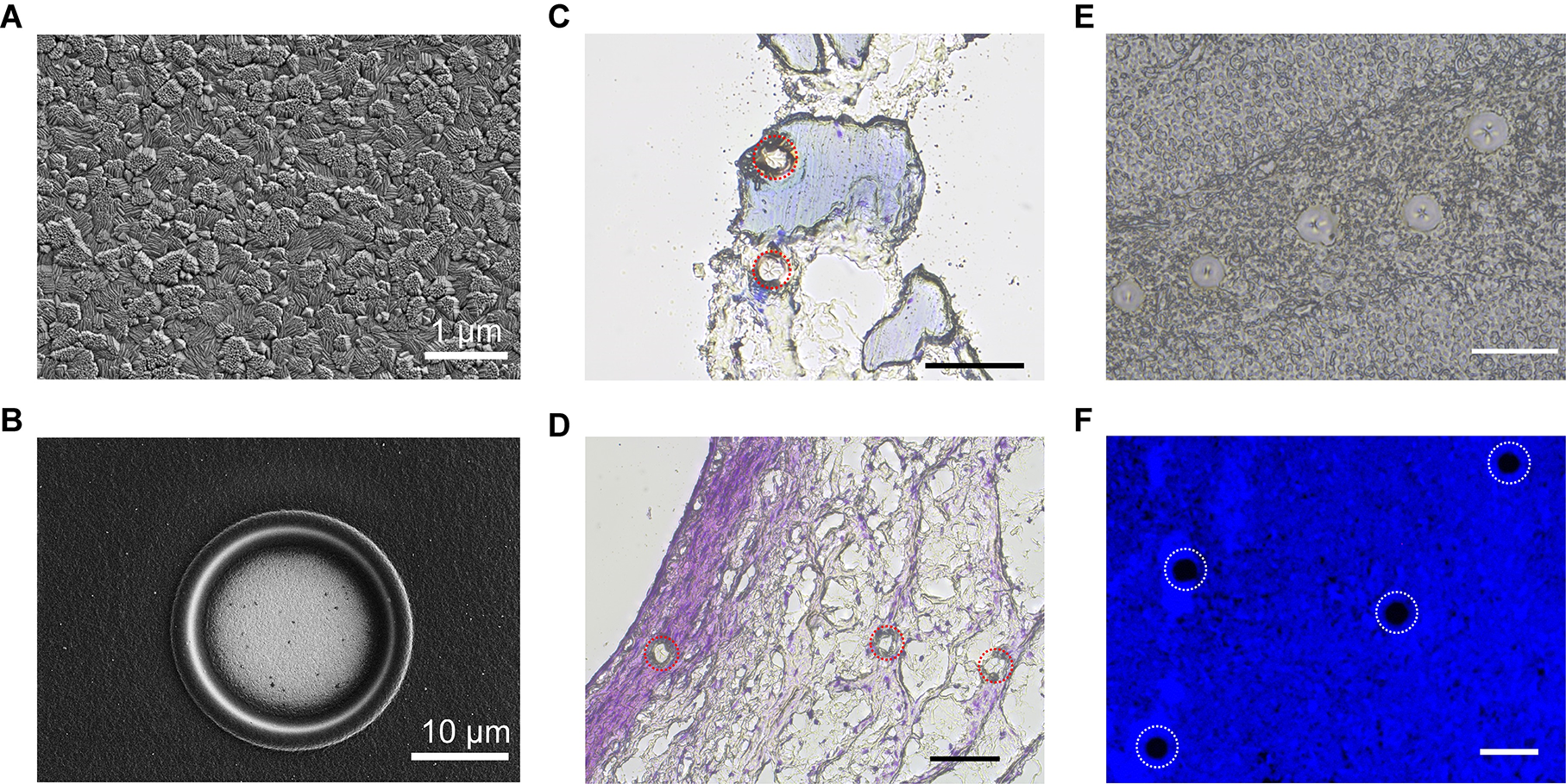

Supplement: S1 Fig — (TIF) [file pone.0305977.s002.tif]

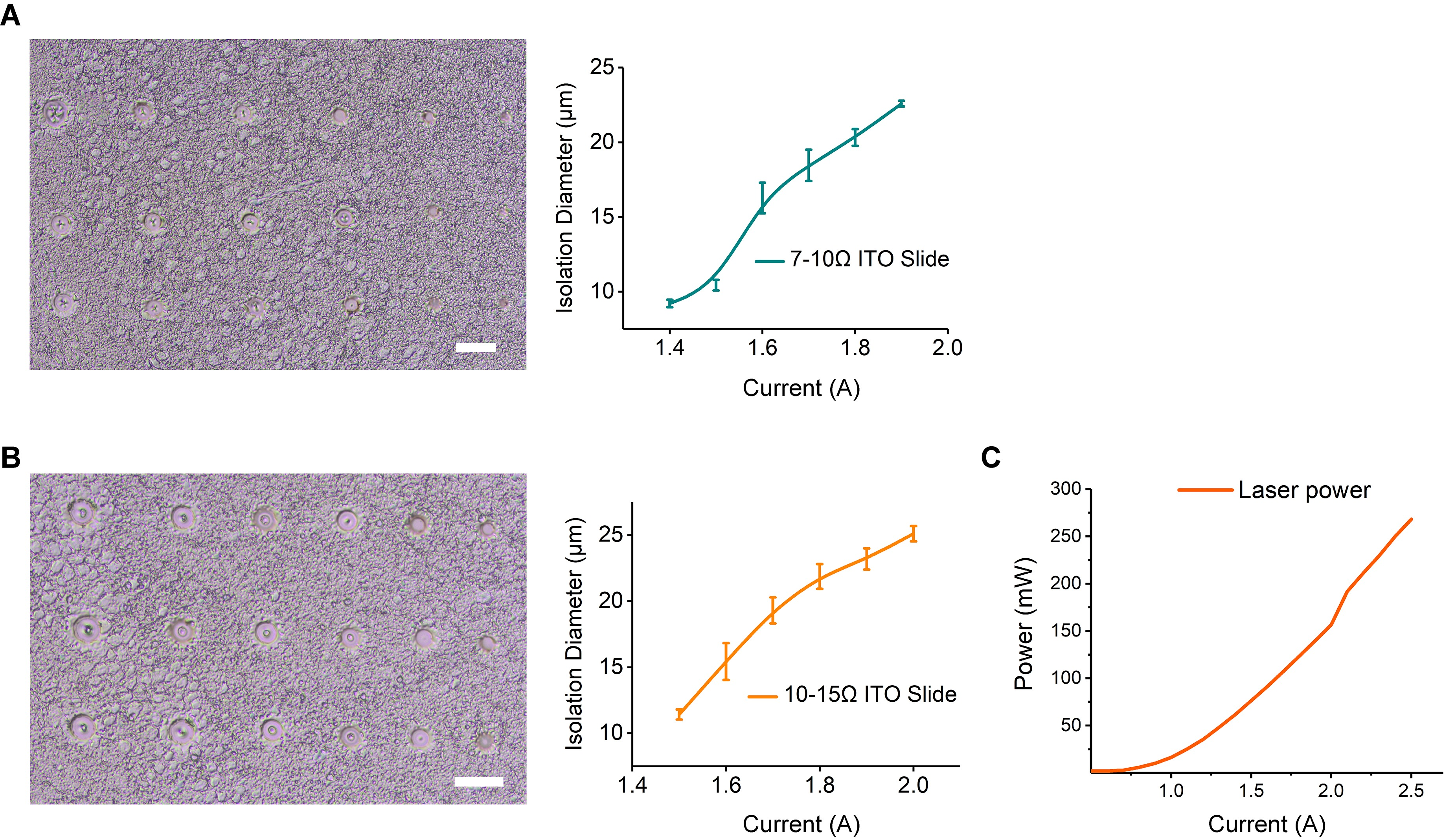

Supplement: S2 Fig — (TIF) [file pone.0305977.s003.tif]

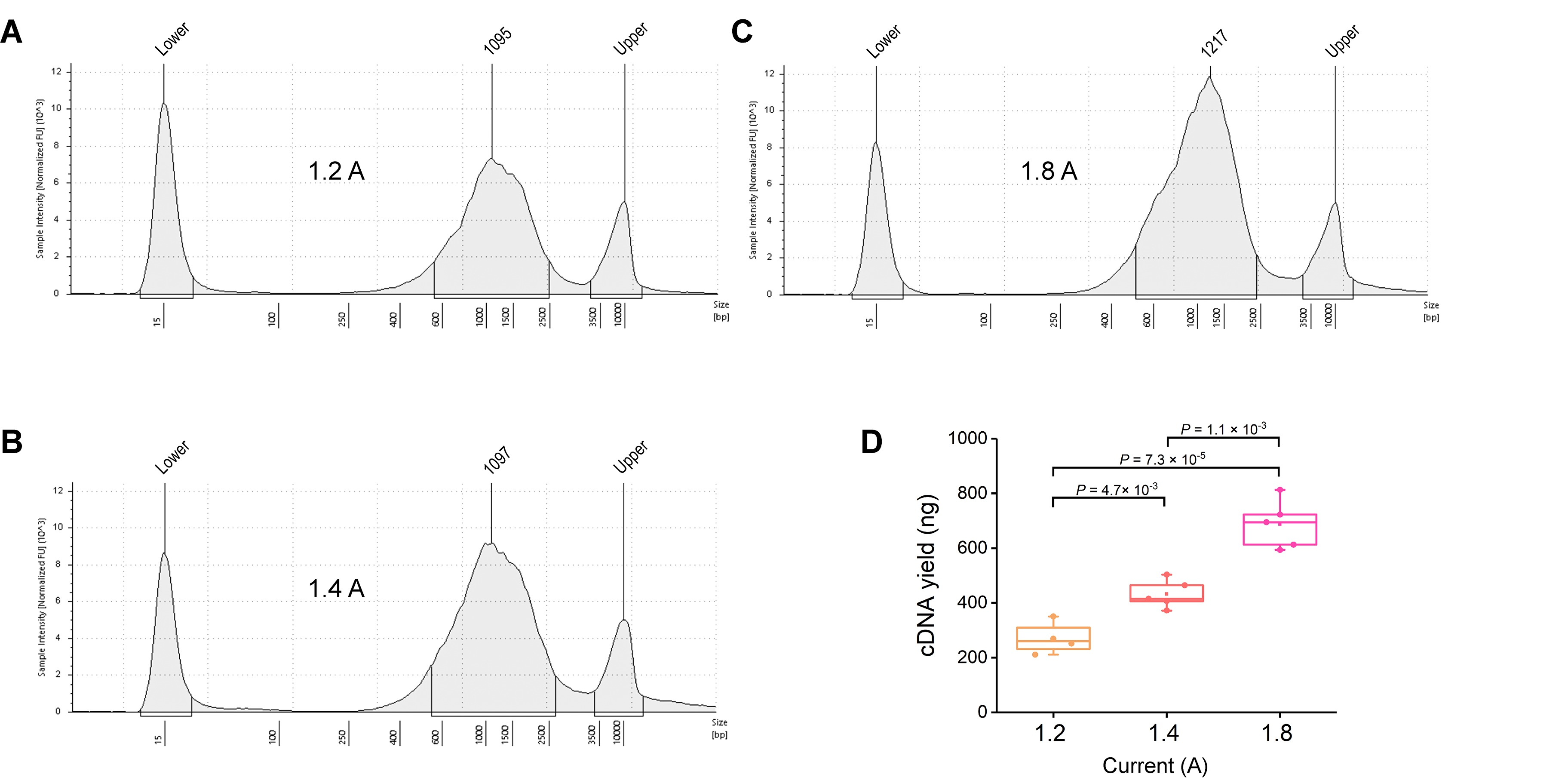

Supplement: S3 Fig — (TIF) [file pone.0305977.s004.tif]

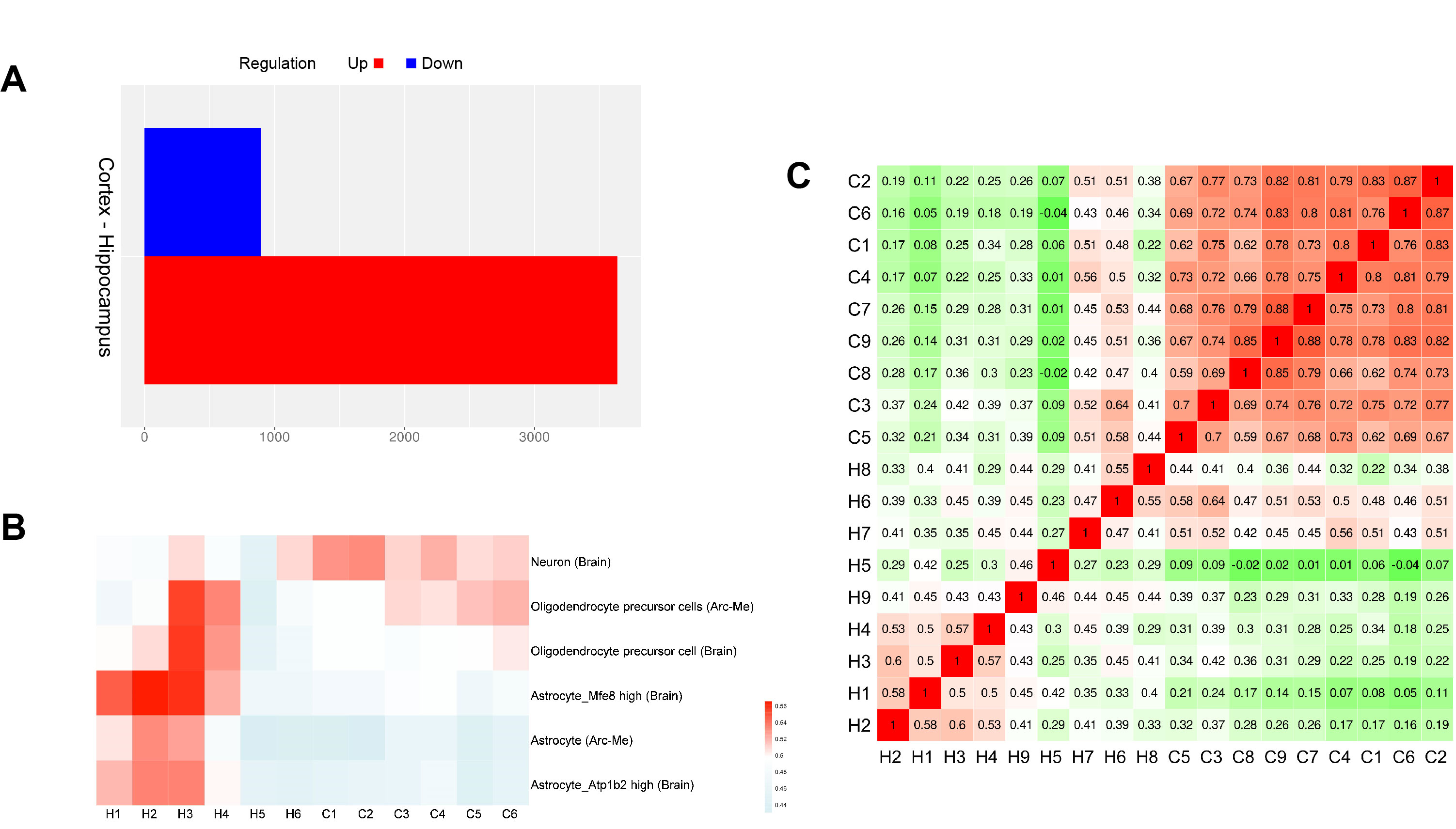

Supplement: S4 Fig — (TIF) [file pone.0305977.s005.tif]

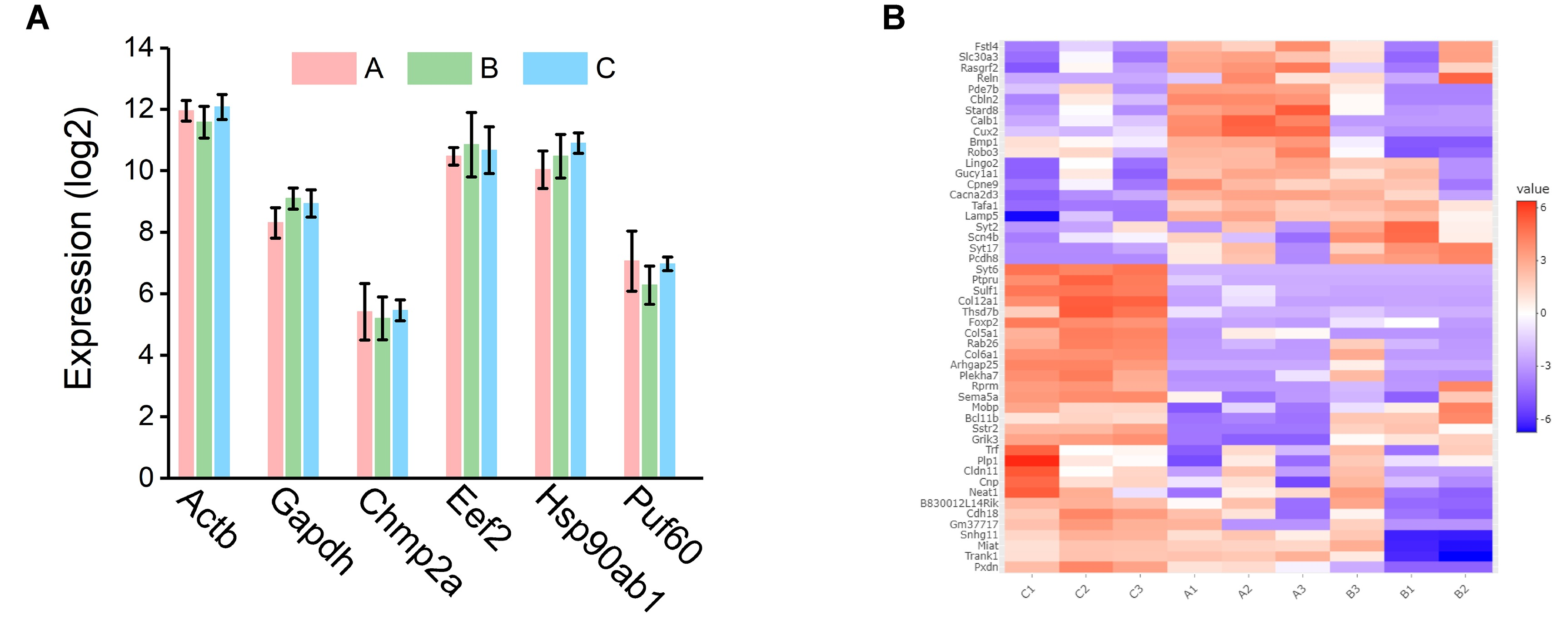

Supplement: S5 Fig — (TIF) [file pone.0305977.s006.tif]
